# Rare Event Analysis for Minimum Hellinger Distance Estimators via Large Deviation Theory

**DOI:** 10.3390/e23040386

**Published:** 2021-03-24

**Authors:** Anand N. Vidyashankar, Jeffrey F. Collamore

**Affiliations:** 1 Department of Statistics, George Mason University, Fairfax, VA 22030, USA; 2 Department of Mathematical Sciences, University of Copenhagen, Universitetsparken 5, DK-2100 Copenhagen Ø, Denmark; collamore@math.ku.dk

**Keywords:** Hellinger distance, large deviations, divergence measures, rare event probabilities

## Abstract

Hellinger distance has been widely used to derive objective functions that are alternatives to maximum likelihood methods. While the asymptotic distributions of these estimators have been well investigated, the probabilities of rare events induced by them are largely unknown. In this article, we analyze these rare event probabilities using large deviation theory under a potential model misspecification, in both one and higher dimensions. We show that these probabilities decay exponentially, characterizing their decay via a “rate function” which is expressed as a convex conjugate of a limiting cumulant generating function. In the analysis of the lower bound, in particular, certain geometric considerations arise that facilitate an explicit representation, also in the case when the limiting generating function is nondifferentiable. Our analysis involves the modulus of continuity properties of the affinity, which may be of independent interest.

## 1. Introduction

In a variety of applications, the use of divergence-based inferential methods is gaining momentum, as these methods provide robust alternatives to traditional maximum likelihood-based procedures. Since the work of [1,2], divergence-based methods have been developed for various classes of statistical models. A comprehensive treatment of these ideas is available, for instance, in [3,4]. The objective of this paper is to study the large deviation tail behavior of the minimum divergence estimators and, more specifically, the minimum Hellinger distance estimators (MHDE).

To describe the general problem, suppose $\Theta \subset R^{d}$, and let $F=\{f_{\theta }\left(\cdot \right):\theta \in \Theta \}$ denote a family of densities indexed by θ. Let $\left\{X_{n}:n\geq 1\right\}$ denote a class of i.i.d. random variables, postulated to have a continuous density with respect to Lebesgue measure and belonging to the family F, and let *X* be a generic element of this class. We denote by g(·) the true density of *X*.

Before providing an informal description of our results, we begin by recalling that the square of the Hellinger distance (SHD) between two densities $h_{1}\left(\cdot \right)$ and $h_{2}\left(\cdot \right)$ on R is given by

$$\begin{array}{c} {\mathrm{HD}}^{2}\left(h_{1},h_{2}\right)={∥h_{1}^{\frac{1}{2}}-h_{2}^{\frac{1}{2}}∥}_{2}^{2}=2-2\int_{R}{\left(h_{1}\left(x\right)h_{2}\left(x\right)\right)}^{\frac{1}{2}}dx. \end{array}$$

The quantity $\int_{R}{\left(h_{1}\left(x\right)h_{2}\left(x\right)\right)}^{\frac{1}{2}}dx$ is referred to as the *affinity* between $h_{1}\left(\cdot \right)$ and $h_{2}\left(\cdot \right)$ and denoted by $A(h_{1},h_{2})$. Hence, the SHD between the postulated density and the true density is given by $\mathrm{SHD}\left(\theta \right)={\mathrm{HD}}^{2}\left(f_{\theta },g\right).$ When Θ is compact, it is known that there exists a unique $\theta_{g}\in \Theta$ minimizing the SHD(θ). Furthermore, when $g\left(\cdot \right)=f_{\theta_{0}}\left(\cdot \right)$ and F satisfies an identifiability condition, it is well known that $\theta_{g}$ coincides with $\theta_{0}$; cf. [1]. Turning to the sample version, we replace g(·) by $g_{n}\left(\cdot \right)$ in the definition of SHD, obtaining the objective function ${\mathrm{SHD}}_{n}\left(\theta \right)={\mathrm{HD}}^{2}\left(f_{\theta },g_{n}\right)$ and

(1) $$\begin{array}{c} g_{n}\left(x\right)=\frac{1}{nb_{n}}\sum_{i=1}^{n}K\left(\frac{x-X_{i}}{b_{n}}\right), \end{array}$$

where the kernel K(·) is a probability density function and $b_{n}↘0$ and $nb_{n}↗\infty$ as n→∞.

It is known that when the parameter space Θ is compact, there exists a unique ${\hat{\theta }}_{n}\in \Theta$ minimizing ${\mathrm{SHD}}_{n}\left(\theta \right)$, and that ${\hat{\theta }}_{n}$ converges almost surely to $\theta_{g}$ as n→∞; cf. [1]. Furthermore, under some natural assumptions,

(2) $$\begin{array}{c} n^{\frac{1}{2}}\left({\hat{\theta }}_{n}-\theta_{g}\right)\overset{d}{\to }G, \end{array}$$

where, under the probability measure associated with g(·), *G* is a Gaussian random vector with mean vector 0 and covariance matrix $\Sigma_{g}$. If $g\left(\cdot \right)=f_{\theta_{0}}\left(\cdot \right)$, then the variance of *G* coincides with the inverse of the Fisher information matrix $I(\theta_{0})$, yielding statistical efficiency. When the true distribution g(·) does not belong to F, we will call this the “model misspecifed case,” while when g∈F, we will say that the “postulated model” holds.

In this paper, we focus on the large deviation behavior of $\left\{{\hat{\theta }}_{n}:n\geq 1\right\}$; namely, the asymptotic probability that the estimate ${\hat{\theta }}_{n}$ will achieve values within a set *away* from the central tendency described in (2). We establish results of the form

(3) $$\begin{array}{c} \log P_{g}\left({\hat{\theta }}_{n}\in B\right)\approx -n\;\underset{\theta \in B}{\inf}I\left(\theta \right), \end{array}$$

for some “rate function” *I* and given Borel subset B⊂Θ. Similar large deviation estimates for maximum likelihood estimators (MLE) have been investigated in [5,6,7], and for general *M*-estimators in [8,9]. These results allow for a precise description of the probabilities of Type I and Type II error in both the Neymann–Pearson and likelihood ratio test frameworks. Furthermore, large deviation bounds allow one to identify the best exponential rate of decrease of Type II error amongst all tests that satisfy a bound on the Type I error, as in Stein’s lemma (cf. [10]). Additional evidence of the importance of large deviation results for statistical inference has been described in [11] and in the book [12].

One of our initial goals was to derive sharp probability bounds for Type I and Type II error in the context of robust hypothesis testing using Hellinger deviance tests. This article is a first step towards this endeavor. A key issue that distinguishes our work from earlier works is that, in our case, the objective function is a nonlinear function of the smoothed empirical measure, and the analysis of this case requires more involved methods compared with those currently existing in the statistical literature on large deviations. Consistent with large deviation analysis more generally, we identify the rate function *I* as the convex conjugate of a certain limiting cumulant generating function, although in our problem, we uncover a subtle asymmetry between the upper and lower bounds when our limiting generating function is nondifferentiable. In the classical large deviation literature, similar asymmetries have been studied in other one-dimensional contexts (e.g. [13]), although the statistical problem is still quite different, as the dependence on the parameter θ arises explicitly—inhibiting the use of convexity methods typically exploited in the large deviation literature—and hence requiring novel techniques.

### 1.1. Large Deviations

In this subsection we provide relevant definitions and properties from large deviation theory required in the sequel. In the following, $R_{+}$ will denote the set of non-negative real numbers.

**Definition** **1.** *A collection of probability distributions $\left\{P_{n}:n\geq 1\right\}$ on a topological space (X,B) is said to satisfy the weak large deviation principle if*

$$\begin{array}{c} \underset{n\to \infty }{lim sup}\frac{1}{n}\log P_{n}\left(F\right)\leq -\underset{x\in F}{\inf}I\left(x\right),\;\mathrm{for}\;\mathrm{all}\;\mathrm{closed}\;F\in B, \end{array}$$

*and*

$$\begin{array}{c} \underset{n\to \infty }{lim inf}\frac{1}{n}\log P_{n}\left(G\right)\geq -\underset{x\in G}{\inf}I\left(x\right)\;\mathrm{for}\;\mathrm{all}\;\mathrm{open}\;\mathrm{sets}\;F\in B \end{array}$$

*for some lower semicontinuous function I:X→[0,∞]. The function I is called the* rate function. *If the level sets of I are compact, we call I a* good rate function *and we say that $\left\{P_{n}\right\}$ satisfies the* large deviation principle *(LDP)*.

We begin with a brief review of large deviation results for i.i.d. random variables and empirical measures. Let $\{X_{n}\}\subset R$ be an i.i.d. sequence of real-valued random variables, and let $P_{n}$ denote the distribution of the sample mean ${\bar{X}}_{n}$. If the moment generating function of $X_{1}$ is finite in a neighborhood of the origin, then Cramér’s theorem states that $\left\{P_{n}\right\}$ satisfies the LDP with good rate function $\Lambda^{*}$, where $\Lambda^{*}$ is the convex conjugate (or Legendre–Fenchel transform) of Λ, and where $\Lambda \left(\alpha \right)=\log E\left[e^{\alpha X_{1}}\right]$ is the cumulant generating function of $X_{1}$ (cf. [10], Section 2.2).

Next, consider the empirical measures $\left\{\mu_{n}\right\}$, defined by

(4) $$\begin{array}{c} \mu_{n}\left(B\right)=\frac{1}{n}\sum_{i=1}^{n}I_{\left\{X_{i}\in B\right\}},\;B\in B, \end{array}$$

where B denotes the collection of Borel subsets of R. It is well known (cf. [14]) that $\left\{\mu_{n}\right\}$ converges weakly to *P*, namely to the distribution of $X_{1}$. Then Sanov’s theorem asserts that $\left\{\mu_{n}\right\}$ satisfies a large deviation principle with rate function $I_{P}$ given by

(5) $$\begin{array}{c} I_{P}\left(\nu \right)=\left\{\begin{array}{cc} \mathrm{KL}(\nu ,P) & \mathrm{if}\;\nu \ll P,\\ \infty & \mathrm{otherwise}, \end{array}\right. \end{array}$$

where KL(ν,P) is the *Kullback–Leibler information* between the probability measures ν and *P*. When ν and *P* each possesses a density with respect to Lebesgue measure (say *p* and *g*, respectively), the above expression becomes

(6) $$\begin{array}{c} \mathrm{KL}\left(p,g\right):=\left\{\begin{array}{cc} \int_{S}p\left(x\right)\log\left(\frac{p(x)}{g(x)}\right)d\mu \left(x\right) & \mathrm{if}\;p\ll g,\\ \infty & \mathrm{otherwise}. \end{array}\right. \end{array}$$

In Sanov’s theorem, the rate function $I_{P}$ is defined on the space of probability measures, which is a metric space with the open sets induced by weak convergence. Extensions of Sanov’s theorem to strong topologies have been investigated in the literature; cf., e.g., [15].

We now turn to a general result, which will play a central role in this paper, namely Varadhan’s integral lemma (cf. [10], Theorem 4.3.1). This result will allow us to infer the scaled limit of a sequence of generating functions from the existence of the large deviation principle.

**Lemma** **1** (Varadhan)**.** *Let $\left\{Y_{n}\right\}$ be a sequence of random variables taking values in a regular topological space (X,B), and assume that the probability law of $\left\{Y_{n}\right\}$ satisfies the LDP with good rate function I. Then for any bounded continuous function F:X→R,*

(7) $$\begin{array}{c} \lim_{n\to \infty }\frac{1}{n}\log E\left[\exp\left(nF\left(Y_{n}\right)\right)\right]=\underset{x\in X}{\sup}\left\{F(x)-I(x)\right\}. \end{array}$$

### 1.2. Minimum Hellinger Distance Estimator and Large Deviations

We first observe that the MHDE is obtained by maximizing

(8) $$\begin{array}{c} A_{n}\left(\theta \right)\equiv A_{n}\left(\theta ,g_{n}\right):=\int_{R}f_{\theta }^{\frac{1}{2}}\left(x\right)g_{n}^{\frac{1}{2}}\left(x\right)d\mu \left(x\right), \end{array}$$

which involves solving the equation $\nabla A_{n}\left(\theta \right)=0.$ The idea behind the large deviation analysis is to observe that the large deviation behavior of the maximizer can be extracted from that of the objective function $\nabla A_{n}\left(\theta \right)$ near 0. By the Gärtner–Ellis theorem (cf. [10], Section 2.3), this amounts to investigating the asymptotic behavior as n→∞ of

(9) $$\begin{array}{c} \frac{1}{a_{n}}\log E_{g}\left[\exp\left\{a_{n}\langle \alpha ,{\nabla A}_{n}\left(\theta \right)\rangle \right\}\right],\;\alpha \in R^{d}, \end{array}$$

where $a_{n}↗\infty$ as n→∞. In the case of maximum likelihood estimation (MLE) or minimum contrast estimation (MCE), the objective function can be expressed as

(10) $$\begin{array}{c} \sum_{i=1}^{n}h_{\theta }\left(X_{i}\right)=n\int_{R}h_{\theta }\left(x\right)d\mu_{n}\left(x\right), \end{array}$$

where $\left\{\mu_{n}:n\geq 1\right\}$ is the empirical measure associated with $\left\{X_{k}:1\leq k\leq n\right\}$. Thus, while the objective functions associated with the MLE and MCE are linear functions of the empirical measure, the affinity is a nonlinear function of the empirical measure. This creates certain complications in identifying the rate function I(·) alluded to in (3). Of course, in the case of likelihood and minimum contrast estimator analysis, an explicit formula for I(·) ensues as the Legendre–Fenchel transform of the cumulant generating function of $h_{\theta }\left(X_{1}\right)$, viz. $\log E_{\theta_{0}}\left[\exp(\alpha h_{\theta }\left(X_{1}\right))\right]$. One approach to evaluating the limiting generating function is to apply Varadhan’s lemma as given above in (7). In the context of our problem, that requires an investigation into the large deviation principle for the density estimators $g_{n}\left(\cdot \right)$ viewed as elements of $L_{1}\left(S\right)$, viz. the space of integrable functions on *S*. Equivalently, we require a version of Sanov’s theorem in $L_{1}$-space, which leads to certain topological considerations. The main issue here is that, when $L_{1}$ is equipped with a norm topology, the sequence of kernel density estimates $\left\{g_{n}\left(\cdot \right)\right\}$ possesses large deviation bounds, but the associated rate function may not have compact level sets, as is required for a typical application of Varadhan’s lemma. Nonetheless, one obtains a full LDP when $L_{1}\left(S\right)$ is equipped with the weak topology.

The asymptotic properties of MHDE, such as consistency and asymptotic normality, are established using the norm convergence of $g_{n}\left(\cdot \right)$ to g(·). For this reason, we focus on a subclass of densities G (see Proposition 1 below) possessing certain equicontinuity properties where norm convergence prevails. These issues are handled in Section 2, where the precise statements of our main results can also be found. Section 3 is devoted to the proofs of the main results. Section 4 contains some concluding remarks.

## 2. Notation, Assumptions, and Main Results

Let $f_{\theta }\left(\cdot \right)$ denote the postulated density of $\left\{X_{n}\right\}$, defined on a measure space (Ω,F). Let S⊂R denote the support of *X* and $s_{\theta }\left(\cdot \right)=f_{\theta }^{\frac{1}{2}}\left(\cdot \right)$. Let the true density of $\left\{X_{n}\right\}$ be given by g(·). Throughout the paper, we assume that the following regularity conditions hold.

**Hypothesis** **1.** *Θ is a compact and convex subset of $R^{d}$.*

**Hypothesis** **2.** *The family F is identifiable; namely, if $\theta_{1}\neq \theta_{2}$, $f_{\theta_{1}}\left(\cdot \right)\neq f_{\theta_{2}}\left(\cdot \right)$ on a set of positive Lebesgue measure.*

**Hypothesis** **3.** *For every θ∈Θ, $s_{\theta }$ is three times continuously differentiable with respect to all components of θ. Denote by $\nabla s_{\theta }$ the gradient of $s_{\theta }$ and its components by ${\dot{s}}_{\theta }^{i}\left(\cdot \right)$. Let $H_{\theta }$ denote the matrix of second partial derivatives of $s_{\theta }\left(\cdot \right)$ with respect to θ and ${\ddot{s}}_{\theta }^{ij}$ the ${\left(i,j\right)}^{th}$ element of $H_{\theta }$.*

**Hypothesis** **4.** *Let the matrix of second partial derivatives of $A_{n}\left(\theta \right)$ and A(θ) be denoted by $H_{A_{n}}\left(\theta \right)$ and $H_{A}\left(\theta \right)$, respectively. Assume that $H_{A_{n}}\left(\theta \right)$ and $H_{A}\left(\theta \right)$ are continuous in θ and that $H_{A}\left(\theta \right)$ is positive definite for every θ∈Θ. For p∈G and θ∈Θ, let $\lambda_{\theta }\left(p\right)$ denote the smallest eigenvalue of the matrix $\int_{S}H_{\theta }\left(x\right)p^{\frac{1}{2}}\left(x\right)dx.$ Assume that $\inf\{\lambda_{\theta }\left(p\right):p\in G\}\geq c>0$, where c is independent of θ.*

These hypotheses on the family F are generally standard and are used to establish the asymptotic properties of the MHDE. Sufficient conditions on F for the validity of these hypotheses are described in [3,16], and [17]. A remark on Hypothesis 4 is warranted here. When p=g, this assumption is related to the positive definiteness of the Fisher information matrix. If one assumes G=F, then this hypothesis reduces to the condition that $\inf\{\lambda_{\theta }:\theta \in \Theta \}\geq c>0$, which is standard. Finally, we remark that we have not attempted to provide the weakest regularity conditions, and we do believe some of these conditions can possibly be relaxed.

Recall that the MHDE of θ can be obtained by solving the equation

(11) $$\begin{array}{c} {\nabla A}_{n}\left(\theta \right)\;:=\nabla_{\theta }A\left(f_{\theta },g_{n}\right)=\frac{1}{2}\int_{R}u_{\theta }\left(x\right)s_{\theta }\left(x\right)g_{n}^{\frac{1}{2}}\left(x\right)dx=0, \end{array}$$

where $u_{\theta }\left(x\right)=\nabla_{\theta }f_{\theta }\left(x\right){\left(f_{\theta }\left(x\right)\right)}^{-1}$ is the *score function*, which is obtained using $\nabla_{\theta }s\left(x;\theta \right)=\frac{1}{2}u\left(x;\theta \right)s\left(x;\theta \right)$.

We begin by providing some heuristics for the case d=1. Let ${\dot{A}}_{n}\left(\theta \right)$ denote the derivative of $A_{n}\left(\theta \right)$ when d=1. Let ${\hat{\theta }}_{n}$ denote the argzero of the function ${\dot{A}}_{n}\left(\theta \right)$ obtained from (11) above. Let ${\hat{\theta }}_{n,l}=\inf\left\{\theta \in \Theta :{\dot{A}}_{n}\left(\theta \right)\leq 0\right\}$ and ${\hat{\theta }}_{n,u}=\sup\left\{\theta \in \Theta :{\dot{A}}_{n}\left(\theta \right)\geq 0\right\}$. Since ${\hat{\theta }}_{n,l}\leq {\hat{\theta }}_{n}\leq {\hat{\theta }}_{n,u}$, we obtain using Markov’s inequality that for any ϵ>0,

(12) $$\begin{array}{c} P_{g}\left({\hat{\theta }}_{n,l}\geq \theta_{g}+\epsilon \right)\leq P_{g}\left({\dot{A}}_{n}\left(\theta_{g}+\epsilon \right)\geq 0\right)\leq E_{g}[\exp\left(n\alpha {\dot{A}}_{n}\left(\theta_{g}+\epsilon \right)\right], \end{array}$$

where α>0. Similarly, for α<0, it can be seen that

(13) $$\begin{array}{c} P_{g}\left({\hat{\theta }}_{n,u}\leq \theta_{g}-\epsilon \right)\leq P_{g}\left({\dot{A}}_{n}\left(\theta_{g}-\epsilon \right)\leq 0\right)\leq E_{g}[\exp\left(n\alpha {\dot{A}}_{n}\left(\theta_{g}-\epsilon \right)\right]. \end{array}$$

Thus, an evaluation of (9) will allow us to obtain the logarithmic upper bound for ${\hat{\theta }}_{n,l}$ and ${\hat{\theta }}_{n,u}$. Next, using the inequalities

(14) $$\begin{array}{ccc} P_{g}\left({\hat{\theta }}_{n,l}\geq \theta_{g}+\epsilon \right) & \leq & P_{g}\left({\dot{A}}_{n}\left(\theta_{g}+\epsilon \right)\geq 0\right)\leq P_{g}\left({\hat{\theta }}_{n,u}\geq \theta_{g}+\epsilon \right), \end{array}$$

(15) $$\begin{array}{ccc} P_{g}\left({\hat{\theta }}_{n,u}\leq \theta_{g}-\epsilon \right) & \leq & P_{g}\left({\dot{A}}_{n}\left(\theta_{g}-\epsilon \right)\leq 0\right)\leq P_{g}\left({\hat{\theta }}_{n,l}\leq \theta_{g}-\epsilon \right), \end{array}$$

under additional hypotheses, one can derive large deviation lower bounds for ${\hat{\theta }}_{n}$. Deriving these bounds for MLE and MCE is rather standard, since the objective functions and their derivatives are *linear* functionals of the empirical distribution, as stated in (10), but this is not the case for the Hellinger distance.

Observe that the probabilities in (12) and (13) represent rare-event probabilities since, under the hypotheses described previously, ${\hat{\theta }}_{n}$ converges to $\theta_{g}$ almost surely as n→∞. The distributional results concerning ${\hat{\theta }}_{n}$ rely on the continuity and differentiability properties of $\nabla A_{n}\left(\theta \right)$, which depend nonlinearly on $g_{n}$, and the norm convergence of $g_{n}$ to *g*.

Let G denote the collection of all probability densities with support *S*. By Scheffe’s theorem, the pointwise convergence of $g_{n}$ to *g* implies $g_{n}\overset{L_{1}}{\to }g$ as n→∞. Additionally, when $g_{n}\left(\cdot \right)$ is the kernel density estimator, then Glick’s Theorem guarantees that $g_{n}\overset{L_{1}}{\to }g$ almost surely as n→∞ when $b_{n}↘0$ and n↗∞; cf. [18]. Since the MHDE are functionals of density estimators, it is natural to expect that the large deviations of density estimators will play a significant role in our analysis. For this reason, one is forced to consider the topological issues that arise in the large deviation analysis of density estimators. Interestingly, it turns out that the weak topology on $L_{1}\left(S\right)$ plays a prominent role. This, in turn, leads to the question of whether certain continuity properties, which were part of the traditional theory of MHD analysis, continue to hold if G were viewed as a subset of $L_{1}\left(S\right)$ equipped with weak topology. Expectedly, while the answer in general is no (cf. [19]), Proposition 1 provides sufficient conditions on the family G under which one additionally obtains norm convergence.

Before proceeding, we now introduce some further regularity conditions, as follows.

**Hypothesis** **5.** *$u_{\theta }s_{\theta }\in L_{2}\left(S\right)$ and is an $L_{2}\left(S\right)$-continuous function of θ.*

**Hypothesis** **6.** *The family F consists of bounded equicontinuous densities.*

**Hypothesis** **7.** *The family G consists of bounded and equicontinuous densities.*

**Hypothesis** **8.** *$u_{\theta }g\in L_{2}\left(S\right)$ and is an $L_{2}\left(S\right)$-continuous function of θ.*

Here, we note that Hypotheses 6 and 7 are related. Furthermore, if one is willing to assume that G=F, then one does not need Hypothesis 7. On the other hand, if one believes that parametric distributions are approximations to G, then one needs to work with Hypothesis 7. For this reason, we have maintained both of these hypotheses in our main results. Hypotheses 5 and 8 are related to finiteness of the Fisher information and are standard in the statistical literature.

Before we state the first proposition, we recall the definition of weak topology on $L_{1}$ (cf. [19]). A sequence $\left\{h_{n}:n\geq 1\right\}$ is said to converge weakly in $L_{1}$ if $\int_{S}h_{n}\left(x\right)b\left(x\right)dx\to \int_{S}h\left(x\right)b\left(x\right)dx$ as n→∞ for every $b\in L_{\infty }\left(S\right)$, where $L_{\infty }\left(S\right)$ is a class of essentially bounded functions. We assume throughout the paper that the topology on Θ is the standard topology generated by the Euclidean metric.

**Proposition** **1.** *Let G denote the class of densities, equipped with the weak topology. Further assume that Hypotheses 1–7 hold. Let Θ⊗G be equipped with the product topology. Then the mapping $\nabla A:\Theta ⊗G\to R^{d}$ defined by*

(16) $$\begin{array}{c} \nabla A\left(\theta ,g\right)\int_{R}u_{\theta }\left(x\right)s_{\theta }\left(x\right)g^{\frac{1}{2}}\left(x\right)dx \end{array}$$

*is jointly continuous in (θ,g). Furthermore, if $g_{n}\overset{w}{\to }g$, then*

(17) $$\begin{array}{c} \lim_{n\to \infty }\underset{\theta \in \Theta }{\sup}\;||\nabla A\left(\theta ,g_{n}\right)-\nabla A\left(\theta ,g\right)\left|\right|=0. \end{array}$$

*Finally, under Hypothesis 7, the family G is a weakly sequentially closed subset of $L_{1}\left(S\right)$.*

Our next result is concerned with the limit behavior of the generating function of ${\nabla A}_{n}\left(\theta \right)$. In the following we use the notation p≪g to mean the probability measures associated with p(·) and g(·) are absolutely continuous.

**Theorem** **1.** *Assume that Hypotheses 1–7 hold, and set*

(18) $$\begin{array}{c} \Lambda_{n,\theta }\left(\alpha \right)\;:=\;\frac{1}{n}\log E_{g}[\exp\left(n\langle \alpha ,{\nabla A}_{n}\left(\theta \right)\rangle \right],\;\alpha \in R^{d}. \end{array}$$

*Then $\Lambda_{\theta }\left(\alpha \right):=\lim_{n\to \infty }\Lambda_{n,\theta }\left(\alpha \right)$ exists and is a convex function given by*

(19) $$\begin{array}{c} \Lambda_{\theta }\left(\alpha \right)=\underset{p\in G}{\sup}\left\{\int_{S}\langle \alpha ,u_{\theta }\left(x\right)\rangle s_{\theta }\left(x\right)p^{\frac{1}{2}}\left(x\right)dx-\mathrm{KL}\left(p,g\right)\right\}, \end{array}$$

*where*

(20) $$\begin{array}{c} \mathrm{KL}\left(p,g\right)=\left\{\begin{array}{cc} \int_{S}p\left(x\right)\log\left(\frac{p(x)}{g(x)}\right)dx & \mathrm{if}\;p\ll g,\\ \infty & \mathrm{otherwise}. \end{array}\right. \end{array}$$

**Remark** **1.** *Since $\Lambda_{\theta }$ is defined via a limiting operation, it is hard to extract its qualitative properties. However, we can obtain a simple lower bound by observing that KL(p,g)=0 if and only if p=g, and an upper bound using that the Kullback–Leibler information is nonnegative. This results in the following bounds:*

(21) $$\begin{array}{c} \int_{S}\langle \alpha ,u_{\theta }\left(x\right)\rangle s_{\theta }\left(x\right)g^{\frac{1}{2}}\left(x\right)dx\leq \Lambda_{\theta }\left(\alpha \right)\leq \underset{p\in G}{\sup}\left[\int_{S}\langle \alpha ,u_{\theta }\left(x\right)\rangle s_{\theta }\left(x\right)p^{\frac{1}{2}}\left(x\right)dx\right]. \end{array}$$

*Furthermore, if all densities in G are bounded by one, then $p^{\frac{1}{2}}\left(\cdot \right)\geq p\left(\cdot \right)$ implies*

(22) $$\begin{array}{ccc} \Lambda_{\theta }\left(\alpha \right) & \geq & \underset{p\in G}{\sup}\left\{\int_{S}\langle \alpha ,u_{\theta }\left(x\right)\rangle s_{\theta }\left(x\right)p\left(x\right)dx-\mathrm{KL}\left(p,g\right)\right\}. \end{array}$$

Using a variational argument, it can be shown that the supremum on the right-hand side is attained at $p^{*}$ given by

(23) $$\begin{array}{c} p^{*}\left(x\right)\;:=\frac{\exp\left(\langle \alpha ,u_{\theta }\left(x\right)\rangle \right)s_{\theta }\left(x\right)}{\int_{S}\langle \alpha ,u_{\theta }\left(x\right)\rangle s_{\theta }\left(x\right)g\left(x\right)dx}; \end{array}$$

cf. [20]. Furthermore, the maximum that results from this choice of $p^{*}\left(\cdot \right)$ is

$$\log\int_{S}\exp\left(\langle \alpha ,u_{\theta }\left(x\right)\rangle \right)s_{\theta }\left(x\right)g\left(x\right)dx,$$

*yielding yet another lower bound for $\Lambda_{\theta }\left(\alpha \right)$, although the comparison of these two lower bounds is not immediate.*

Returning to our main discussion, recall from [21] that the convex conjugate of the function $\Lambda_{\theta }$ is defined by

(24) $$\begin{array}{c} \Lambda_{\theta }^{*}\left(x\right)=\underset{\alpha \in R^{d}}{\sup}\left\{\langle \alpha ,x\rangle -\Lambda (\alpha )\right\},\;x\in R^{d}. \end{array}$$

Let $D_{\theta }$ denote the domain of $\Lambda_{\theta }$; namely,

(25) $$\begin{array}{c} D_{\theta }=\left\{\alpha \in R^{d}:\Lambda_{\theta }\left(\alpha \right)<\infty \right\}; \end{array}$$

and let $R_{\theta }$ denote the range of the gradient map $\nabla \Lambda_{\theta }$; that is,

$$R_{\theta }=\left\{x\in R^{d}:\nabla \Lambda_{\theta }\left(\alpha \right)=x,\;\mathrm{some}\;\alpha \in R^{d}\right\}.$$

We begin with the discussion of the case d=1. In this case, the generating function $\Lambda_{\theta }$ reduces to

(26) $$\begin{array}{c} \Lambda_{\theta }\left(\alpha \right)=\underset{p\in G}{\sup}\left\{\alpha \int_{S}\exp(n\alpha {\dot{A}}_{n}\left(\theta \right)s\left(x;\theta \right)p^{\frac{1}{2}}\left(x\right)dx-\mathrm{KL}\left(p,g\right)\right\}. \end{array}$$

By the convexity of $\Lambda_{\theta }\left(\cdot \right)$, this function is differentiable almost everywhere (cf. [21]), and in the proof, we would like to exploit the differentiability of this function at the point $\alpha_{\theta }^{*}$ where it attains its minimum value. If $\Lambda_{\theta }$ is not differentiable at this point, it is helpful to consider the directional derivatives of $\Lambda_{\theta }$. Specifically, let $\Lambda_{\theta ,+}^{\prime }\left(\cdot \right)$ and $\Lambda_{\theta ,-}^{\prime }\left(\cdot \right)$ denote the right and left derivatives of $\Lambda_{\theta }\left(\cdot \right)$, respectively. When $x\in \left(\Lambda_{\theta ,-}^{\prime }\left(\alpha \right),\Lambda_{\theta ,+}^{\prime }\left(\alpha \right)\right)$, then it is well known that $\Lambda_{\theta }^{*}\left(x\right)=\alpha x-\Lambda_{\theta }\left(\alpha \right)$, but this observation will not be sufficient to obtain a proper lower bound. For that to hold, we need a stronger condition, namely that $0\in R_{\theta }$, which will only be true if $\Lambda_{\theta }$ is differentiable at its point of minimum, $\alpha_{\theta }^{*}$. Otherwise, the expected lower bound turns out to be $\Lambda_{\theta }^{*}\left(x\right)$, where $x=\Lambda_{\theta ,+}^{\prime }\left(\alpha_{\theta }^{*}\right)$; cf. [13].

We now turn to our large deviation theorem in $R^{1}$, where we study the rare-event probabilities $P_{g}\left({\hat{\theta }}_{n}\in C\right)$ for sets *C* that are away from the true value $\theta_{g}$. Specifically, we establish an analogue of the LDP, but where a subtle difference arises in the lower bound in the absence of differentiability of $\Lambda_{\theta }$.

We recall that ${\hat{\theta }}_{n}$ is defined using the kernel density estimator $g_{n}\left(\cdot \right)$ defined in (1), whose behavior is dictated by the bandwidth sequence $\left\{b_{n}\right\}$.

**Theorem** **2.** *Assume d=1, Hypotheses 1–8 are satisfied, and ${\hat{\theta }}_{n}$ is the unique zero of ${\dot{A}}_{n}\left(\theta \right)=0$. Further assume that $b_{n}↘0$ and $nb_{n}↗\infty$ as n→∞. Then for any closed set F not containing $\theta_{g}$,*

(27) $$\begin{array}{c} \underset{n\to \infty }{lim sup}\frac{1}{n}\log P_{g}\left({\hat{\theta }}_{n}\in F\right)\leq -\underset{\theta \in F}{\inf}\Lambda_{\theta }^{*}\left(0\right). \end{array}$$

*Moreover, for any open set G not including $\theta_{g}$,*

(28) $$\begin{array}{c} \underset{n\to \infty }{lim inf}\frac{1}{n}\log P_{g}\left({\hat{\theta }}_{n}\in G\right)\geq -\underset{\theta \in G}{\inf}I\left(\theta \right), \end{array}$$

*where*

(29) $$\begin{array}{c} I\left(\theta \right)=\inf\left\{\Lambda_{\theta }^{*}\left(x\right):x\in R_{\theta }\cap \left[0,\infty \right)\right\}, \end{array}$$

*and the infimum is taken to be infinity if the set $R_{\theta }\cap \left[0,\infty \right)$ is empty.*

**Remark** **2.** *If F=[θ,∞) where $\theta >\theta_{g}$, then in both the upper and lower bounds, it is sufficient to evaluate the infimum at the boundary point θ. That is,*

$$\begin{array}{c} \underset{n\to \infty }{lim sup}\frac{1}{n}\log P_{g}\left({\hat{\theta }}_{n}\in \left[\theta ,\infty \right)\right)\leq -\Lambda_{\theta }^{*}\left(0\right). \end{array}$$

*Similarly, if G=(θ,∞) where $\theta >\theta_{g}$, then*

$$\begin{array}{c} \underset{n\to \infty }{lim inf}\frac{1}{n}\log P_{g}\left({\hat{\theta }}_{n}\in \left(\theta ,\infty \right)\right)\geq -I\left(\theta \right). \end{array}$$

Furthermore, if $\inf_{\alpha }\Lambda_{\theta }\left(\alpha \right)$ is achieved at a unique point $\alpha_{\theta }^{*}$ and $\Lambda_{\theta }$ is differentiable at $\alpha_{\theta }^{*}$, then the right-hand side of (28) reduces to $\Lambda_{\theta }^{*}\left(0\right)$, i.e., the upper and lower bounds coincide and the limits exist. Since the rate function appearing in the upper and lower bounds coincide in this case, we obtain a proper LDP *if* the resulting rate function has the required regularity properties, in particular, $I\left(\theta \right)=\Lambda_{\theta }^{*}\left(0\right)$ is lower semicontinuous and has compact level sets.

The proof of the above theorem relies on (14) and (15) combined with Theorem 1, together with a change of measure argument characteristic of large deviation analysis. The comparison inequalities in (14) and (15) are critical to obtaining the characterizations in the above theorem, but these are essentially one-dimensional results and their analogues in higher dimensions (d≥2) are not immediate. Consequently, when $\Lambda_{\theta }$ is not differentiable, new complications arise, which lead to a slightly different, and less explicit, representation of the lower bound.

Next we establish a large deviation theorem for $R^{d}$, generalizing the previous theorem to higher dimensions. In the following, let $\mathrm{dist}\left(x,G\right)=\inf_{y\in G}\left||x-y|\right|$ denote the distance between a point $x\in R^{d}$ and a set $G\subset R^{d}$.

**Theorem** **3.** *Assume Hypotheses 1–8 are satisfied, and assume that $b_{n}↘0$ and $nb_{n}↗\infty$ as n→∞. Then for any closed set F not containing$\;\theta_{g}$,*

(30) $$\begin{array}{c} \underset{n\to \infty }{lim sup}\frac{1}{n}\log P_{g}\left({\hat{\theta }}_{n}\in F\right)\leq -\underset{\theta \in F}{\inf}\Lambda_{\theta }^{★}\left(0\right). \end{array}$$

*Moreover, for any open set G not including $\theta_{g}$,*

(31) $$\begin{array}{c} \underset{n\to \infty }{lim inf}\frac{1}{n}\log P_{g}\left({\hat{\theta }}_{n}\in G\right)\geq -\underset{\theta \in G}{\inf}I\left(\theta \right), \end{array}$$

where $I\left(\theta \right)=\inf\left\{\Lambda_{\theta }^{*}\left(x\right):x\in R_{\theta }\cap B\left(0;c_{\theta }\right)\right\}$ and $c_{\theta }=b\;\mathrm{dist}\left(\theta ,\Theta -G\right)$ for some universal constant b∈(0,∞), and the infimum is taken to be infinity if the set $R_{\theta }\cap B\left(0;c_{\theta }\right)$ is empty.

**Remark** **3.** As we noted for the one-dimensional case in Remark 2, under a differentiability assumption on $\Lambda_{\theta }$, the function I(θ) can be identified as $\Lambda_{\theta }^{*}\left(0\right)$, but in full generality, it is not immediately known that I(θ) is even nontrivial. Moreover, without differentiability, the infimum in the definition of I(θ) is *more* restrictive than what we encountered in the one-dimensional problem. However, if one assumes additional geometry on *G*, such as a translated cone structure, then one obtains improved estimates in the sense that one can take unbounded regions in the definition of I(θ), just as we saw in Theorem 2.2. For further remarks in this direction, see the discussion given after the proof of the theorem.

## 3. Proofs

We turn first to Proposition 1.

**Proof** **of** **Proposition** **1.** Since Θ⊗G is equipped with product topology, it is sufficient to show that if $\theta_{n}\to \theta$ and $g_{n}\overset{w}{\to }g$, then ${\nabla A}_{n}\left(\theta \right)$ converges to ∇A(θ), where

(32) $$\begin{array}{c} \nabla A\left(\theta \right)=\int_{S}u_{\theta }\left(x\right)s_{\theta }\left(x\right)g^{\frac{1}{2}}\left(x\right)dx. \end{array}$$

Let $r_{\theta }\left(x\right)=u_{\theta }\left(x\right)s_{\theta }\left(x\right)$, and observe that

(33) $$\begin{array}{ccc} \left|\nabla A\left(\theta_{n},g_{n}\right)-\nabla A\left(\theta ,g\right)\right| & \leq & \int_{S}|r_{\theta_{n}}\left(x\right)||g_{n}^{\frac{1}{2}}\left(x\right)-g^{\frac{1}{2}}\left(x\right)|dx+\int_{S}\left|r_{\theta_{n}}\left(x\right)-r_{\theta }\left(x\right)\right|g^{\frac{1}{2}}\left(x\right)dx\\ & \leq & \left|\right|r_{\theta }{\left|\right|}_{2}\mathrm{HD}\left(g_{n},g\right)+\int_{S}\left|r_{\theta_{n}}\left(x\right)-r_{\theta }\left(x\right)\right|g^{\frac{1}{2}}\left(x\right)dx\\ & = & T_{n,1}+T_{n,2}, \end{array}$$

where the penultimate equation follows by applying the Cauchy–Schwarz inequality. Then by the Cauchy–Schwarz inequality and Hypothesis 5, $T_{n,2}\to 0$. Since Hellinger distance is dominated by the $L_{1}$-distance, in order to complete the proof, it is sufficient to show that $\left|\right|g_{n}-g{\left|\right|}_{1}\to 0$. Now since $g_{n}\overset{w}{\to }g$, it follows that as n→∞,

(34) $$\begin{array}{c} G_{n}\left(x\right)\;:=\int_{S}g_{n}\left(y\right)I_{\left\{y\leq x\right\}}dy\to \int_{S}g\left(y\right)I_{\left\{y\leq x\right\}}dyG\left(x\right). \end{array}$$

Evidently, $G_{n}\left(\cdot \right)$ and G(·) are nondecreasing and right continuous. Furthermore, if $x_{*}=\inf\left\{x:x\in S\right\}$ and $x^{*}=\sup\left\{x:x\in S\right\}$, then $G_{n}\left(x_{*}\right)\to G\left(x_{*}\right)$ and $G_{n}\left(x^{*}\right)\to G\left(x^{*}\right)$, where $G_{n}\left(x_{*}\right)=\lim_{x\to x_{*}}G_{n}\left(x\right)$, $G_{n}\left(x^{*}\right)=\lim_{x\to x*}G_{n}\left(x\right)$, $G\left(x_{*}\right)=\lim_{x\to x_{*}}G\left(x\right)$, $G\left(x^{*}\right)=\lim_{x\to x*}G\left(x\right)$. Thus $G_{n}$ converges to *G*, which is a proper distribution function. Then by Lemma 1 of Boos [22], $g_{n}\left(\cdot \right)$ converges to g(·) uniformly on compact sets. This, in turn, implies the $L_{1}$ convergence of $g_{n}\left(\cdot \right)$ to g(·) (by Scheffe’s lemma), which establishes the convergence of $T_{n,1}$ to 0, thus completing the proof of the joint continuity of ∇A(θ,g).

Next, the uniform convergence (17) follows by Hypothesis 5, since

$$\begin{array}{ccc} \underset{\theta \in \Theta }{\sup}\left|\nabla A\left(\theta ,g_{n}\right)-\nabla A\left(\theta ,g\right)\right| & \leq & \int_{S}|r_{\theta }\left(x\right)||g_{n}^{\frac{1}{2}}\left(x\right)-g^{\frac{1}{2}}\left(x\right)|dx\\ & \leq & \underset{\theta \in \Theta }{\sup}\left|\right|r_{\theta }{\left|\right|}_{2}\mathrm{HD}\left(g_{n},g\right)\to 0. \end{array}$$

Finally, to prove that G is weakly sequentially closed, note that convergence in weak topology implies pointwise convergence, yielding g(·)≥0. Noting that

(35) $$\begin{array}{c} \int_{S}g\left(x\right)d\mu \left(x\right)=1+\int_{S}\left(g\left(x\right)-g_{n}\left(x\right)\right)dx, \end{array}$$

it follows that g(·) integrates to one, using $L_{1}$ convergence, thus completing the proof of the proposition. □

We now turn to the proof of Theorem 1. The proof relies on the large deviation theorem for the kernel density estimator $g_{n}\left(\cdot \right)$ in the weak topology of G. The next proposition is concerned with the LDP for $\left\{g_{n}\right\}$ in G, equipped with the inherited weak topology from $L_{1}\left(S\right)$. This issue has received considerable attention recently (cf. [23,24]), where it is established that the full LDP may *not* hold for $\left\{g_{n}\right\}$ in norm topology, but does hold under the weak topology.

**Proposition** **2.** *Assume Hypotheses 1–8 and that $b_{n}↘0$ and $nb_{n}↗\infty$ as n→∞. Then $\left\{g_{n}\right\}$ satisfies the LDP in the weak topology of $L_{1}\left(S\right)$ with good rate function I given by*

(36) $$\begin{array}{c} I\left(p\right)=\left\{\begin{array}{cc} \int_{S}p\left(x\right)\log\left(\frac{p(x)}{g(x)}\right)dx & \mathrm{if}\;g\ll p,\\ \infty & \mathrm{otherwise}. \end{array}\right. \end{array}$$

**Proof** **of** **Theorem** **1.** As before, let G be equipped with the weak topology. Set $r_{\theta }\left(x\right)=u_{\theta }\left(x\right)s_{\theta }\left(x\right)$, and define F:G→R as follows:

(37) $$\begin{array}{c} F\left(h\right)=\int_{S}\langle \alpha ,r_{\theta }\left(x\right)\rangle h^{\frac{1}{2}}\left(x\right)dx. \end{array}$$

By Hypothesis 5, $r_{\theta }\in L_{2}\left(S\right)$. To show that F(·) is continuous, let $h_{n}\overset{w}{\to }h$ as n→∞. Then

(38) $$\begin{array}{ccc} |F\left(h_{n}\right)-F\left(h\right)| & \leq & \int_{S}r_{\theta }\left(x\right)\left|h_{n}^{\frac{1}{2}}\left(x\right)-h^{\frac{1}{2}}\left(x\right)\right|d\mu \left(x\right)\\ & \leq & \left|\right|r_{\theta }{\left|\right|}_{2}\mathrm{HD}\left(h_{n},h\right)\leq \left|\right|r_{\theta }{\left|\right|}_{2}\left|\right|h_{n}-h{\left|\right|}_{1}\to 0\;\mathrm{as}\;n\to \infty , \end{array}$$

where we have used the Cauchy–Schwarz inequality that the $L_{1}$ distance dominates the Hellinger distance in (38). Now by Hypothesis 7, as in the proof of Proposition 1, we have that $\left|\right|h_{n}-h{\left|\right|}_{1}\to 0$ as n→∞, establishing the continuity of F(·). Next, to show that F(·) is bounded, note that $\sup\left\{F\left(p\right):p\in G\right\}\leq \left|\right|r_{\theta }{\left|\right|}_{2}$ by the Cauchy–Schwarz inequality. Then by Proposition 2, it follows by Varadhan’s integral lemma (see [10], Theorem 4.3.1) that

(39) $$\begin{array}{ccc} \lim_{n\to \infty }\frac{1}{n}\log E\left[\exp(nF\left(g_{n}\left(x\right)\right)\right] & = & \lim_{n\to \infty }\frac{1}{n}\log E\left[\exp\left(n\int_{S}\langle \alpha ,u_{\theta }\left(x\right)s_{\theta }\left(x\right)\rangle g_{n}^{\frac{1}{2}}\left(x\right)dx\right)\right]\\ & = & \underset{p\in G}{\sup}\left\{\int_{S}\langle \alpha ,u_{\theta }\left(x\right)\rangle s_{\theta }\left(x\right)p^{\frac{1}{2}}\left(x\right)dx-\mathrm{KL}\left(p,g\right)\right\}\\ & :=\; & \Lambda_{\theta }\left(\alpha \right). \end{array}$$

This completes the proof of the theorem. □

The proofs of our main results will involve probability bounds on the modulus of continuity of $A_{n}\left(\theta \right)$ and ${\nabla A}_{n}\left(\theta \right)$, respectively. Recall that the modulus of continuity ω(h;r) of a function $h:R^{d}\to R$ is given by

(40) $$\begin{array}{c} \omega (h;r)\;:=\;\underset{\left|\right|x_{1}-x_{2}\left|\right|\leq r}{\sup}\left|h\left(x_{1}\right)-h\left(x_{2}\right)\right|,\;r>0. \end{array}$$

Observe that when h(·) is replaced by $A_{n}\left(\theta \right)$ or ${\nabla A}_{n}\left(\theta \right)$, the modulus of continuity becomes a random quantity. Our next proposition summarizes the continuity properties of $A_{n}\left(\theta \right)$ and ${\nabla A}_{n}\left(\theta \right)$ via their modulus of continuity as real-valued functionals from G equipped with the weak topology.

**Proposition** **3.** *Assume that Hypotheses 1–8 hold and that $b_{n}↘0$ and $nb_{n}↗\infty$ as n→∞. Then, with respect to $\left\{A_{n}\right\}$ and A, the modulus of continuity satisfies the following relations, each with probability one:*

$$\begin{array}{c} \left(i\right)\;\lim_{n\to \infty }\omega \left(A_{n};r\right)=\omega \left(A,r\right);\;\left(\mathrm{ii}\right)\;\lim_{r\to 0}\omega \left(A_{n};r\right)=0;\;\mathrm{and}\;\left(\mathrm{iii}\right)\;\lim_{r\to 0}\omega \left(A;r\right)=0. \end{array}$$

*Similarly, the sequence $\left\{\nabla A_{n}\right\}$ and ∇A satisfy the analogous relations with probability one; namely,*

$$\begin{array}{c} \left(\mathrm{iv}\right)\;\lim_{n\to \infty }\omega \left(\nabla A_{n};r\right)=\omega \left(\nabla A;r\right);\;\left(v\right)\;\lim_{r\to 0}\omega \left(\nabla A_{n};r\right)=0;\;\mathrm{and}\;\left(\mathrm{vi}\right)\;\lim_{r\to 0}\omega \left(\nabla A;r\right)=0. \end{array}$$

**Proof.**  First observe that $A_{n}\left(\theta \right)$ converges uniformly to A(θ). To see this, note that if $g_{n}\overset{w}{\to }g$, then by Proposition 1, it converges in $L_{1}$. Hence

(41) $$\begin{array}{ccc} \underset{\theta \in \Theta }{\sup}\left|A_{n}\left(\theta \right)-A\left(\theta \right)\right| & \leq & \underset{\theta \in \Theta }{\sup}\int_{R}s_{\theta }\left(x\right)\left|g_{n}^{\frac{1}{2}}\left(x\right)-g^{\frac{1}{2}}\left(x\right)\right|dx\\ & \leq & \left|\right|g_{n}^{\frac{1}{2}}\left(x\right)-g^{\frac{1}{2}}{\left(x\right)||}_{2}\leq \left|\right|g_{n}-g{\left|\right|}_{1}\to 0, \end{array}$$

where the last inequality follows using that the Hellinger distance is dominated by the $L_{1}$-distance. We now prove (i). For this we invoke the properties of the modulus of continuity. Observe that

(42) $$\begin{array}{c} \omega \left(A_{n};r\right)=\omega \left(A_{n}-A+A;r\right)\leq \omega \left(A_{n}-A;r\right)+\omega \left(A;r\right), \end{array}$$

which yields

(43) $$\begin{array}{c} |\omega \left(A_{n};r\right)-\omega \left(A;r\right)|\leq \omega \left(A_{n}-A;r\right). \end{array}$$

Next observe that

(44) $$\begin{array}{ccc} \omega (A_{n}-A;r) & = & \underset{\left|\right|\theta_{1}-\theta_{2}\left|\right|\leq r}{\sup}\left|\left(A_{n}-A\right)\left(\theta_{1}\right)-\left(A_{n}-A\right)\left(\theta_{2}\right)\right|\\ & \leq & 2\underset{\theta \in \Theta }{\sup}\left|A_{n}\left(\theta \right)-A\left(\theta \right)\right|\to 0, \end{array}$$

where the last convergence follows from the uniform convergence of $\left(A_{n}-A\right)\left(\theta \right)$ to 0 as shown in (42). The proof of (iv) is similar, and specifically is obtained by using that

(45) $$\begin{array}{c} \omega \left(\nabla \left(A_{n}-A\right);r\right)\leq 2\underset{\theta \in \Theta }{\sup}||\nabla A_{n}\left(\theta \right)-\nabla A\left(\theta \right)\left|\right|\to 0, \end{array}$$

where the above convergence follows from (17).

We now turn to the proof of (ii). Using the Cauchy–Schwarz inequality and the definition of Hellinger distance,

(46) $$\begin{array}{ccc} \omega (A_{n};r) & = & \underset{\left|\right|\theta_{1}-\theta_{2}\left|\right|\leq r}{\sup}\left|A_{n}\left(\theta_{1}\right)-A_{n}\left(\theta_{2}\right)\right|\\ & = & \underset{\left|\right|\theta_{1}-\theta_{2}\left|\right|\leq r}{\sup}\left|\int_{R}\left(s_{\theta_{1}}\left(x\right)-s_{\theta_{2}}\left(x\right)\right)g_{n}^{\frac{1}{2}}\left(x\right)dx\right|\leq \mathrm{HD}\left(f_{\theta_{1}},f_{\theta_{2}}\right)\\ & \leq & \underset{\left|\right|\theta_{1}-\theta_{2}\left|\right|\leq r}{\sup}\left|\right|f_{\theta_{1}}-f_{\theta_{2}}{\left|\right|}_{1}\;:=\;\omega \left(H;r\right), \end{array}$$

where $H:\left(\theta_{1},\theta_{2}\right)\to \left|\right|f_{\theta_{1}}-f_{\theta_{2}}{\left|\right|}_{1}$ is continuous since F is continuous in θ. Also, since Θ×Θ is compact, H(·,·) is uniformly continuous. Since the modulus of continuity converges to 0 if and only if H(·,·) is uniformly continuous, (ii) follows. Turning to (v), notice that, as before,

(47) $$\begin{array}{c} \omega \left(\nabla A_{n};r\right)\leq \underset{\left|\right|\theta_{1}-\theta_{2}\left|\right|\leq r}{\sup}\left|\right|u_{\theta_{1}}s_{\theta_{1}}-u_{\theta_{2}}s_{\theta_{2}}{\left|\right|}_{2}. \end{array}$$

Now, since $u_{\theta }s_{\theta }$ is $L_{2}$ continuous, by Hypothesis 5, the proof follows as in (ii) due to to the compactness of Θ. The proofs of (iii) and (vi) are similar to (ii) and (v), respectively, and are therefore omitted. □

**Proposition** **4.** *For any 0<M<∞ and δ>0, there exists a positive number r(M,δ) such that*

(48) $$\begin{array}{c} P_{g}\left(\omega \left(A_{n};r\right)\geq \delta \right)\leq e^{-Mn}\;and\;P_{g}\left(\omega \left(\nabla A_{n};r\right)\geq \delta \right)\leq e^{-Mn}. \end{array}$$

**Proof.**  By Markov’s inequality and (46), it follows that for any β>0,

(49) $$\begin{array}{c} P_{g}\left(\omega \left(A_{n};r\right)\geq \delta \right)\leq E_{g}\left[e^{n\beta \omega (A_{n};r)}\right]e^{-n\beta \delta }\leq e^{-n\beta (\delta -\omega (H;r))}. \end{array}$$

Since ω(H;r)→0 as r↘0, there exists an $r_{0}$ such that for all $r\leq r_{0}$, (δ−ω(H;r))>0. Since β>0 is arbitrary, the proposition follows by taking $\beta =M{\left(\delta -\omega \left(H;r\right)\right)}^{-1}$, for some $r\leq r_{0}$. The proof of the second inequality is similar, using (47). □

**Proof** **of** **Theorem** **2.** We begin with the proof of the upper bound. Since we assume that the equation ${\dot{A}}_{n}\left(\theta \right)=0$ has a unique solution, it follows from the inequality in (12) that for any α>0 and $\theta >\theta_{g}$,

(50) $$\begin{array}{c} \underset{n\to \infty }{lim sup}\frac{1}{n}\log P_{g}\left({\hat{\theta }}_{n}\geq \theta \right)\leq \underset{n\to \infty }{lim sup}\frac{1}{n}\log E_{g}\left[\exp\left(n\alpha {\dot{A}}_{n}\left(\theta \right)\right)\right]=\Lambda_{\theta }\left(\alpha \right), \end{array}$$

where the last equality follows by applying Theorem 1 with d=1. Since the inequality holds for every α>0,

(51) $$\begin{array}{ccc} \underset{n\to \infty }{lim sup}\frac{1}{n}\log P_{g}\left({\hat{\theta }}_{n}\geq \theta \right) & \leq & \underset{\alpha >0}{\sup}\Lambda_{\theta }\left(\alpha \right)\leq \underset{\alpha \in R}{\sup}\Lambda_{\theta }\left(\alpha \right). \end{array}$$

Now, noticing that $\sup_{\alpha \in R}\Lambda_{\theta }\left(\alpha \right)=-\inf_{\alpha \in R}-\Lambda_{\theta }\left(\alpha \right)=-\Lambda_{\theta }^{*}\left(0\right)$, we then obtain

(52) $$\begin{array}{c} \underset{n\to \infty }{lim sup}\frac{1}{n}\log P_{g}\left({\hat{\theta }}_{n}\geq \theta \right)\leq -\Lambda_{\theta }^{*}\left(0\right). \end{array}$$

Similarly, for $\theta <\theta_{g}$, using (13), one can show by an analogous calculation that

(53) $$\begin{array}{ccc} \underset{n\to \infty }{lim sup}\frac{1}{n}\log P_{g}\left({\hat{\theta }}_{n}\leq \theta \right) & \leq & -\Lambda_{\theta }^{*}\left(0\right). \end{array}$$

Now let $\theta_{1}=\inf\left\{\theta >\theta_{g}:\theta \in F\right\}$ and $\theta_{2}=\sup\left\{\theta <\theta_{g}:\theta \in F\right\}$. Then

(54) $$\begin{array}{c} P_{g}\left({\hat{\theta }}_{n}\in F\right)\leq P_{g}\left({\hat{\theta }}_{n}\geq \theta_{1}\right)+P_{g}\left({\hat{\theta }}_{n}\leq \theta_{2}\right), \end{array}$$

and so by (52) and (53), it follows that

(55) $$\begin{array}{c} \underset{n\to \infty }{lim sup}\frac{1}{n}\log P_{g}\left({\hat{\theta }}_{n}\in F\right)\leq -\min_{\theta \in \{\theta_{1},\theta_{2}\}}\Lambda_{\theta }^{*}\left(0\right)\leq -\underset{\theta \in F}{\inf}\Lambda_{\theta }^{*}\left(0\right), \end{array}$$

where the last step follows since *F* closed implies $\{\theta_{1},\theta_{2}\}\subset F$.

Next we turn now to the proof of the lower bound. Let *G* be an open set, and let θ∈G. Then there exists an ϵ>0 (to be chosen) such that $I_{\epsilon }\;:=\;(\theta -\epsilon ,\theta +\epsilon )⊊G$. Note that

$$\begin{array}{ccc} \left\{{\hat{\theta }}_{n}\in I_{\epsilon }\right\} & = & \left\{{\dot{A}}_{n}\left({\hat{\theta }}_{n}\right)=0,\;{\hat{\theta }}_{n}\in I_{\epsilon }\right\}\\ & ⊃ & \left\{{\dot{A}}_{n}\left(\theta \right)-{\dot{A}}_{n}\left({\hat{\theta }}_{n}\right)\geq \delta \right\}\cup \{\;{\hat{\theta }}_{n}\in I_{\epsilon },\underset{\theta_{1},\theta_{2}\in I_{\epsilon }}{\sup}|{\dot{A}}_{n}\left(\theta_{1}\right)-{\dot{A}}_{n}\left(\theta_{2}\right)\left|\leq \delta \right\}. \end{array}$$

Thus,

(56) $$\begin{array}{ccc} P_{g}\left({\hat{\theta }}_{n}\in I_{\epsilon }\right) & \geq & P_{g}\left({\dot{A}}_{n}\left(\theta \right)-{\dot{A}}_{n}\left({\hat{\theta }}_{n}\right)\geq \delta \right)-P_{g}\left({\hat{\theta }}_{n}\notin I_{\epsilon },\underset{\theta_{1},\theta_{2}\in I_{\epsilon }}{\sup}|{\dot{A}}_{n}\left(\theta_{1}\right)-{\dot{A}}_{n}\left(\theta_{2}|>\delta \right)\right)\\ & \geq & P_{g}\left({\dot{A}}_{n}\left(\theta \right)-{\dot{A}}_{n}\left({\hat{\theta }}_{n}\right)\geq \delta \right)-P_{g}(\underset{\theta_{1},\theta_{2}\in I_{\epsilon }}{\sup}|{\dot{A}}_{n}\left(\theta_{1}\right)-{\dot{A}}_{n}\left(\theta_{2}\right)\left|>\delta \right)\\ & = & P_{g}\left({\dot{A}}_{n}\left(\theta \right)\geq \delta \right)-P_{g}(\underset{\theta_{1},\theta_{2}\in I_{\epsilon }}{\sup}|{\dot{A}}_{n}\left(\theta_{1}\right)-{\dot{A}}_{n}\left(\theta_{2}\right)\left|>\delta \right)\\ & = & P_{g}\left({\dot{A}}_{n}\left(\theta \right)\geq \delta \right)-P_{g}\left(\omega \left({\dot{A}}_{n};\epsilon \right)>\delta \right). \end{array}$$

We now investigate $P_{g}\left({\dot{A}}_{n}\left(\theta \right)\geq \delta \right)$. Let $Q_{n}$ denote the distribution of ${\dot{A}}_{n}\left(\theta \right)$, and define $Q_{n,\alpha }$ as follows:

(57) $$\begin{array}{c} Q_{n,\alpha }\left(B\right)=\frac{1}{\Lambda_{n,\theta }\left(\alpha \right)}\int_{B}e^{-n\alpha y}dQ_{n}\left(y\right),\;B\in B. \end{array}$$

Let B=(x−η,x+η), for some η>0, where B⊂(δ,∞) and $x\in R_{\theta }$. Then

(58) $$\begin{array}{c} Q_{n}\left(B\right)\geq \exp\left\{-n\alpha x-n\eta |\alpha |+n\Lambda_{n,\theta }\left(\alpha \right)\right\}Q_{n,\alpha }\left(B\right). \end{array}$$

Taking the logarithm, dividing by *n*, and then taking the limit as n→∞, we obtain

(59) $$\begin{array}{c} \underset{n\to \infty }{lim inf}\frac{1}{n}\log Q_{n}\left(B\right)\geq -\alpha x-\eta \left|\alpha \right|-\Lambda_{\theta }\left(\alpha \right)+\underset{n\to \infty }{lim inf}\frac{1}{n}\log Q_{n,\alpha }\left(B\right). \end{array}$$

Now since $x\in R_{\theta }$, we can apply Theorem IV.1 of [25] to obtain that the last term on the right-hand side of the previous equation converges to zero. Upon letting η→0, it follows that

(60) $$\begin{array}{c} \underset{n\to \infty }{lim inf}\frac{1}{n}\log Q_{n}\left(B\right)\geq -\Lambda_{\theta }^{*}\left(x\right). \end{array}$$

Since the above inequality holds for all $x\in R_{\theta }\cap \left(\delta ,\infty \right)$, we conclude that

(61) $$\begin{array}{c} \lim_{n\to \infty }\frac{1}{n}\log P_{g}\left({\dot{A}}_{n}\left(\theta \right)\geq \delta \right)\geq -I_{\delta }\left(\theta \right), \end{array}$$

where $I_{\delta }\left(\theta \right)=\inf_{x\in R_{\theta }\cap \left(\delta ,\infty \right)}\Lambda_{\theta }^{*}\left(x\right)$.

By Proposition 4, choosing $M>I_{\delta }\left(\theta \right)$, one can find ϵ>0 such that

(62) $$\begin{array}{c} P_{g}\left(\omega \left({\dot{A}}_{n};\epsilon \right)>\delta \right)\leq e^{-Mn}. \end{array}$$

Since

(63) $$\begin{array}{c} P_{g}\left({\hat{\theta }}_{n}\in G\right)\geq P_{g}\left({\dot{A}}_{n}\left(\theta \right)\geq \delta \right)\left(1-\frac{P_{g}\left(\omega \left({\dot{A}}_{n};\epsilon \right)\right)}{P_{g}\left({\dot{A}}_{n}\left(\theta \right)\geq \delta \right)}\right), \end{array}$$

by the choice of *M*, it follows from (61) that

(64) $$\begin{array}{c} \underset{n\to \infty }{lim inf}\frac{1}{n}\log P_{g}\left({\hat{\theta }}_{n}\in G\right)\geq -I_{\delta }\left(\theta \right). \end{array}$$

Taking the supremum on left- and right-hand side over all δ>0 yields the required lower bound. □

Turning to the higher dimensional case, we first need the following result, which provides a uniform bound on the Hessian of the objective function $A_{n}\left(\theta \right)$.

**Lemma** **2.** *Under Hypotheses 1–8, there exists a finite constant 0<C<∞ such that with probability one,*

(65) $$\begin{array}{c} \underset{n\geq 1}{\sup}\underset{\theta \in \Theta }{\sup}\;||H_{A_{n}}\left(\theta \right){\left|\right|}_{2}\leq C. \end{array}$$

**Proof.**  This is standard. Specifically, note that the ${\left(i,j\right)}^{\mathrm{th}}$ element of the matrix $H_{A_{n}}\left(\theta \right)$ is given by

(66) $$\begin{array}{c} h_{n,ij}=\int_{S}{\ddot{s}}_{\theta }^{ij}\left(x\right)g_{n}^{\frac{1}{2}}\left(x\right)dx. \end{array}$$

Next, writing down the expression for ${\ddot{s}}_{\theta }^{ij}$ in terms of the derivatives of the score function $u_{\theta }$, using the Cauchy–Schwarz inequality along with Hypotheses 3, 4, 6, and 8, and the definition of the matrix norm, the lemma follows. □

In the proof of the lower bound, we will take a somewhat different approach, involving the analysis of *k* constraints, and our strategy will be to reduce this to a problem involving a single constraint. Specifically, in (67) below, we establish that, instead of studying k constraints on a quantity $D_{n}$ (which we are about to define), we can cast the problem in terms of a *d*-dimensional vector $Y_{n}$ (defined in (70) below) belonging to a ball centered at 0 and of appropriate radius.

To be more precise, let $G\subset R^{d}$ be open, and consider the probability that we obtain an estimated value θ∈G. Let $\{\theta_{1},\ldots ,\theta_{k}\}\subset \Theta -G$, and for any δ>0, set

$$d_{n}\left(j\right)=A_{n}\left(\theta \right)-A_{n}\left(\theta_{j}\right)-\delta ,\;j=1,\ldots ,k$$

and $D_{n}\left(\theta \right)=\left(d_{n}\left(1\right),\cdots d_{n}\left(k\right)\right)$. If θ is chosen as the estimate, then we must have $A_{n}\left(\theta \right)-A_{n}\left(\theta_{j}\right)\geq 0$ for all *j*, so, in particular,

(67) $$\begin{array}{c} P_{g}\left({\hat{\theta }}_{n}\in G\right)\geq P_{g}\left(D_{n}\left(\theta \right)\geq 0\right) \end{array}$$

(by which we mean that $d_{n}\left(j\right)\geq 0$ for all *j* in this last probability).

To evaluate the latter probability, observe that by a second-order Taylor expansion,

(68) $$\begin{array}{c} s_{\theta }\left(x\right)-s_{\theta_{j}}\left(x\right)=\langle \theta -\theta_{j},\nabla s_{\theta }\left(x\right)\rangle +\frac{1}{2}\left(\theta -\theta_{j}\right)H\left(x;\theta_{j}^{*}\right){\left(\theta -\theta_{j}\right)}^{\prime }. \end{array}$$

Using the positive definiteness and uniform boundedness of the matrix $\int_{R}H\left(x;\theta \right)p^{\frac{1}{2}}\left(x\right)dx$, by Hypothesis 4, we have that for any unit vector $v\in R^{d}$,

$$\underset{p\in G}{\sup}\underset{\eta \in \Theta }{\inf}\left\{v\left(\int_{R}H\left(x;\eta \right)p^{\frac{1}{2}}\left(x\right)dx\right)v^{\prime }\right\}\geq c,$$

where *c* is a positive constant independent of v. Thus, for each *j*,

(69) $$\begin{array}{c} \underset{p\in G}{\sup}\underset{\eta \in \Theta }{\inf}\left\{\left(\theta -\theta_{j}\right)\left(\int_{R}H\left(x;\eta \right)p^{\frac{1}{2}}\left(x\right)dx\right){\left(\theta -\theta_{j}\right)}^{\prime }\right\}\geq c\;{∥\theta -\theta_{j}∥}^{2}. \end{array}$$

Integrating with respect to $g_{n}^{\frac{1}{2}}\left(\cdot \right)$ and using the definition of $A_{n}\left(\cdot \right)$, we then obtain that

(70) $$\begin{array}{c} d_{n}\left(j\right)=\int_{R}\left[\langle \theta -\theta_{j},\nabla s(x,\theta )\rangle \right]g_{n}^{\frac{1}{2}}\left(x\right)dx+R\left(\theta ,\theta_{j}\right), \end{array}$$

where

$$R\left(\theta ,\theta_{j}\right)\geq c\;{∥\theta -\theta_{j}∥}^{2}-\delta .$$

Let $Y_{n}\left(\theta \right)=\left(Y_{n,1},\ldots ,Y_{n,d}\right)$, where for $s\left(x;\theta \right):=s_{\theta }\left(x\right)$:

(71) $$\begin{array}{c} Y_{n,j}=\int_{S}\frac{\partial }{\partial \theta_{j}}s\left(x;\theta \right)g_{n}^{\frac{1}{2}}\left(x\right)dx,\;1\leq j\leq k. \end{array}$$

(We have suppressed θ in the notation for $Y_{n,j}$.) Then the inequality $d_{n}\left(j\right)\geq 0$ corresponds to an event $E_{n,j}$ described by the occurrence of the inequality

(72) $$\langle \frac{\theta -\theta_{j}}{||\theta -\theta_{j}\left|\right|},Y_{n}\rangle \geq -c||\theta -\theta_{j}\left|\right|+\delta (||\theta -\theta_{j}{\left||\right)}^{-1},$$

where the right-hand side is always negative for small δ (since dist(θ,Θ−G)>0) and behaves like a constant multiple of dist(θ,Θ−G) as this distance tends to infinity. Thus, we can choose a positive constant $a_{\delta }$ such that

$$a_{\delta }\;\mathrm{dist}\left(\theta ,\Theta -G\right)\leq c||\theta -\theta_{j}\left|\right|-\delta (||\theta -\theta_{j}{\left||\right)}^{-1},\;j=1,\ldots ,k,$$

and set $c_{\theta }\left(\delta \right):=a_{\delta }\;\mathrm{dist}\left(\theta ,\Theta -G\right)$. Finally, let ${\tilde{E}}_{n}$ denote the event that

(73) $$\begin{array}{c} \langle \frac{\theta -\theta_{j}}{||\theta -\theta_{j}\left|\right|},Y_{n}\rangle \geq -c_{\theta }\left(\delta \right). \end{array}$$

Then for all *j*, $E_{n,j}⊃{\tilde{E}}_{n}$, where we recall that $E_{n,j}$ was defined via (72). Now, since the definition of the event ${\tilde{E}}_{n}$ does not depend on any specific vector $\theta_{j}$, one can replace the vector $\left(\theta -\theta_{j}\right)(||\theta -\theta_{j}{\left||\right)}^{-1}$ by any unit vector v in $R^{d}$. Hence

(74) $$\begin{array}{c} P_{g}\left(D_{n}\geq 0\right)\geq P_{g}\left(\langle v,Y_{n}\rangle \geq -c_{\theta }\left(\delta \right),\;\mathrm{for}\;\mathrm{all}\;\mathrm{unit}\;\mathrm{vectors}\;v\right)=P_{g}\left(Y_{n}\in \bar{B}\left(0;c_{\theta }\left(\delta \right)\right)\right), \end{array}$$

and we now derive a large deviation lower bound for the probability on the right-hand side.

**Proposition** **5.** *Assume that Hypotheses 1–8 hold, and suppose that G is an open subset of $R^{d}$. Assume that $b_{n}↘0$ and $nb_{n}↗\infty$ as n→∞. Then for any θ∈G and r>0,*

(75) $$\begin{array}{c} \lim_{n\to \infty }\frac{1}{n}\log P_{g}\left(Y_{n}\in B\left(0;r\right)\right)\geq -I_{r}\left(\theta \right), \end{array}$$

where $I_{r}\left(\theta \right)=\inf\left\{\Lambda_{\theta }^{*}\left(x\right):x\in R_{\theta }\cap B\left(0;r\right)\right\}$ and the infimum is taken to be infinity if the set $R_{\theta }\cap B\left(0;r\right)$ is empty.

**Proof.**  We begin by studying the limiting generating function of $Y_{n}$. By Varadhan’s integral lemma, it follows that

(76) $$\begin{array}{c} \lim_{n\to \infty }\Lambda_{n,\theta }\left(\alpha \right)\;:=\;\lim_{n\to \infty }\frac{1}{n}\log E_{g}[\exp\left(n\langle \alpha ,Y_{n}\rangle \right]=\Lambda_{\theta }\left(\alpha \right), \end{array}$$

where

(77) $$\begin{array}{c} \Lambda_{\theta }\left(\alpha \right)=\underset{p\in G}{\sup}\left[\int_{S}\langle \alpha ,\nabla s_{\theta }\left(x\right)\rangle p^{\frac{1}{2}}\left(x\right)dx-\mathrm{KL}\left(p,g\right)\right]. \end{array}$$

Define the α-shifted distribution by

(78) $$\begin{array}{c} Q_{n,\alpha }\left(B\right)=\frac{1}{\Lambda_{n,\theta }\left(\alpha \right)}\int_{B}e^{n\langle \alpha ,y\rangle }dQ_{n}\left(y\right), \end{array}$$

where $Q_{n}$ denotes the distribution of $Y_{n}$. Note by the convexity of $\Lambda_{\theta }\left(\alpha \right)$ that it is almost everywhere differentiable. Fix $x\in R_{\theta }\cap B\left(0;r\right)$ and choose α such that $\nabla \Lambda_{\theta }\left(\alpha \right)=x$. Let δ>0 be such that B(x;δ)⊊B(0;r). Then

(79) $$\begin{array}{ccc} Q_{n}\left(B\left(x;\delta \right)\right) & = & \exp\left(n\Lambda_{n,\theta }\left(\alpha \right)\right)\int_{B(x;\delta )}\exp(-n\langle \alpha ,y\rangle )dQ_{n,\alpha }\left(y\right)\\ \; & \geq & \exp\left(n(-\langle \alpha ,x\rangle +\Lambda_{n,\theta }\left(\alpha \right)+||\alpha ||\delta )\right)Q_{n,\alpha }\left(B\left(x;\delta \right)\right), \end{array}$$

implying

(80) $$\begin{array}{c} \underset{n\to \infty }{lim inf}\frac{1}{n}\log Q_{n}\left(B\left(x;\delta \right)\right)\geq -\langle \alpha ,x\rangle +\Lambda_{\theta }\left(\alpha \right)-||\alpha ||\delta +\underset{n\to \infty }{lim inf}\frac{1}{n}\log Q_{n,\alpha }\left(B\left(x;\delta \right)\right). \end{array}$$

Now, notice that the limiting cumulant generating function of $Y_{n}$ under the measure $Q_{n,\alpha }$ is given by

(81) $$\begin{array}{c} {\tilde{\Lambda }}_{\theta }\left(\beta \right)=\Lambda_{\theta }\left(\alpha +\beta \right)-\Lambda_{\theta }\left(\beta \right). \end{array}$$

Since ${\tilde{\Lambda }}_{\theta }$ is a proper convex function, it is continuous since $\Lambda_{\theta }\left(\alpha \right)$ is finite in the $R^{d}$, and moreover, by the choice of x, it is differentiable at 0. Hence Condition II.1 of [25] is satisfied. Now, using Theorem IV.1 of [25], it follows that

(82) $$\begin{array}{c} \underset{n\to \infty }{lim inf}\frac{1}{n}\log Q_{n,\alpha }\left(B\left(x;\delta \right)\right)=0. \end{array}$$

Substituting the above into (80), we obtain

(83) $$\begin{array}{c} \underset{n\to \infty }{lim inf}\frac{1}{n}\log P_{g}\left(Y_{n}\in B\left(0;r\right)\right)\geq -\Lambda_{\theta }^{*}\left(x\right). \end{array}$$

Taking the supremum in $x\in R_{\theta }\cap B\left(0;r\right)$, the proposition follows. □

**Proof** **of** **Theorem** **3:** **Upper** **Bound.** Let *F* be a closed subset of Θ. Note Θ compact implies that *F* is compact. Let {B(θ;r):θ∈Θ} denote an open cover of *F*, and let $\left\{B\left(\theta_{1};r\right),\ldots ,B\left(\theta_{k};r\right)\right\}$ denote the finite subcover. Using that $\nabla A_{n}\left({\hat{\theta }}_{n}\right)=0$, we then obtain that for any $\alpha \in R^{d}$,

(84) $$\begin{array}{ccc} P_{g}\left({\hat{\theta }}_{n}\in F\right) & \leq & \sum_{j=1}^{k}P_{g}\left({\hat{\theta }}_{n}\in B\left(\theta_{k};r\right)\right)\\ & = & \sum_{j=1}^{k}E_{g}\left[\exp\left(n\langle \alpha ,{\dot{A}}_{n}\left({\hat{\theta }}_{n}\right)\rangle \right)I_{\left\{{\hat{\theta }}_{n}\in B\left(\theta_{j};r\right)\right\}}\right]\;:=\;\sum_{j=1}^{k}T_{n}\left(j\right). \end{array}$$

Adding and subtracting $\nabla A_{n}\left(\theta_{j}\right)$ to $\nabla A_{n}\left(\theta \right)$ and then applying Hölder’s inequality yields $T_{n}\left(j\right)\leq T_{n}\left(1,j,p\right)T_{n}\left(2,j,q\right)$, where

$$\begin{array}{ccc} \log T_{n}\left(1,j,p\right) & = & \frac{1}{p}\log E_{g}\left[\exp(np{\langle \alpha ,\nabla A}_{n}\left(\theta_{j}\right)\rangle \right)I_{\left\{\theta \in B(\theta_{j};r)\right\}}],\\ \log T_{n}\left(2,j,q\right) & = & \frac{1}{q}\log E_{g}\left[\exp(nq\langle \alpha ,{\nabla (A}_{n}\left({\hat{\theta }}_{n}\right)-A_{n}\left(\theta_{j}\right))\rangle \right)I_{\left\{{\hat{\theta }}_{n}\in B\left(\theta_{j};r\right)\right\}}]. \end{array}$$

First we study $T_{n}\left(2,j,q\right)$. For ${\hat{\theta }}_{n}\in B\left(\theta_{j},r_{j}\right)$ and $\theta_{1},\theta_{2}\in \Theta$, the Cauchy–Schwarz inequality gives

$$\begin{array}{ccc} \left|\langle \alpha ,\nabla A_{n}\left({\hat{\theta }}_{n}\right)-\nabla A_{n}\left(\theta_{j}\right))\rangle \right| & \leq & {\left||\alpha |\right|}_{2}\underset{\theta_{1},\theta_{2}\in B\left(\theta_{j},r\right)}{\sup}||\nabla A_{n}\left(\theta_{1}\right)-\nabla A_{n}\left(\theta_{2}\right){)||}_{2}\\ & \leq & {\left||\alpha |\right|}_{2}\left|r\right|\underset{\theta \in B(\theta_{j},r)}{\sup}\left|\right|H_{A_{n}}\left(\theta \right){\left|\right|}_{2}\\ & \leq & {\left||\alpha |\right|}_{2}\left|r\right|\max_{1\leq j\leq k}\left[\underset{\theta \in B(\theta_{j},r_{j})}{\sup}\left|\right|H_{A_{n}}\left(\theta \right){\left|\right|}_{2}\right], \end{array}$$

where $H_{A_{n}}\left(\theta \right)$ is the Hessian matrix consisting of the second partial derivatives of $A_{n}\left(\theta \right)$. Hence we obtain for any 1≤j≤k that

(85) $$\begin{array}{ccc} \frac{1}{n}\log T_{n}\left(2,j,q\right) & \leq & r\frac{1}{nq}{\left(nq||\alpha |\right|}_{2})\max_{1\leq j\leq k}\left\{\underset{\theta \in B(\theta_{j},r)}{\sup}\left|\right|H_{A_{n}}\left(\theta \right){\left|\right|}_{2}\right\}\\ & = & {r||\alpha ||}_{2}\max_{1\leq j\leq k}\left\{\underset{\theta \in B(\theta_{j},r)}{\sup}\left|\right|H_{A_{n}}\left(\theta \right){\left|\right|}_{2}\right\}. \end{array}$$

Now by Lemma 2,

(86) $$\begin{array}{c} \underset{n\to \infty }{lim sup}\frac{1}{n}\log T_{n}\left(2,j,q\right)\leq Cr. \end{array}$$

Also, for each 1≤j≤k, Theorem 1 provides that

(87) $$\begin{array}{c} \underset{n\to \infty }{lim sup}\frac{1}{n}\log T_{n}\left(1,j,p\right)\leq \frac{1}{p}\Lambda_{\theta_{j}}\left(p\alpha \right). \end{array}$$

Thus

(88) $$\begin{array}{ccc} \underset{n\to \infty }{lim sup}\frac{1}{n}P_{g}\left({\hat{\theta }}_{n}\in F\right) & \leq & \max_{1\leq j\leq k}\underset{n\to \infty }{lim sup}\frac{1}{n}\log T_{n}\left(1,j,p\right)+\max_{1\leq j\leq k}\underset{n\to \infty }{lim sup}\frac{1}{n}\log T_{n}\left(2,j,p\right)\\ & \leq & \max_{1\leq j\leq k}\frac{1}{p}\Lambda_{\theta_{j}}\left(p\alpha \right)+Cr. \end{array}$$

Since the last inequality holds for all p>1,

(89) $$\begin{array}{ccc} \underset{n\to \infty }{lim sup}\frac{1}{n}P_{g}\left({\hat{\theta }}_{n}\in F\right) & \leq & \max_{1\leq j\leq k}\frac{1}{p}\Lambda_{\theta_{j}}\left(p\alpha \right)+Cr\\ & \to & \max_{1\leq j\leq k}\Lambda_{\theta_{j}}\left(\alpha \right)+Cr\;\mathrm{as}\;p↘0. \end{array}$$

Moreover, for each *j*,

$$\begin{array}{c} \Lambda_{\theta_{j}}\left(\alpha \right)\leq \underset{\alpha \in R^{d}}{\sup}\Lambda_{\theta_{j}}\left(\alpha \right):=-\Lambda_{\theta_{j}}\left(0\right). \end{array}$$

Hence

(90) $$\begin{array}{ccc} \underset{n\to \infty }{lim sup}\frac{1}{n}P_{g}\left({\hat{\theta }}_{n}\in F\right) & \leq & \max_{1\leq j\leq k}-\Lambda_{\theta_{j}}^{*}\left(0\right)+Cr\\ & \leq & -\underset{\theta \in F}{\inf}\Lambda_{\theta }^{*}\left(0\right)+Cr. \end{array}$$

The upper bound follows by letting r↘0. □

**Proof** **of** **Theorem** **3:** **Lower** **Bound.** Let *G* be an open subset of Θ, and let θ∈G. Then $G^{c}=\Theta -G$ is compact, and there exists a collection $T=\left\{\theta_{1},\ldots ,\theta_{k}\right\}\subset G^{c}$ such that $B\left(\theta_{1};\epsilon \right),\ldots ,B\left(\theta_{k};\epsilon \right)$ forms a finite subcover of Θ−G, where ϵ>0. Since

(91) $$\begin{array}{ccc} \left\{A_{n}\left(\theta \right)\geq \underset{t\in T}{\sup}A_{n}\left(t\right)\right\} & ⊃ & \left\{A_{n}\left(\theta \right)\geq \max_{1\leq j\leq k}A_{n}\left(\theta_{j}\right)\;\;+\;\max_{1\leq j\leq k}\underset{t\in B(\theta_{j};\epsilon )}{\sup}\left[A_{n}\left(t\right)-A_{n}\left(\theta_{j}\right)\right]\right\}\\ & ⊃ & \left\{A_{n}\left(\theta \right)\geq \max_{1\leq j\leq k}A_{n}\left(\theta_{j}\right)+\;\underset{\left|\right|\theta_{1}-\theta_{2}\left|\right|<\epsilon }{\sup}\left[A_{n}\left(\theta_{1}\right)-A_{n}\left(\theta_{2}\right)\right]\right\} \end{array}$$

it follows that

(92) $$\begin{array}{ccc} P_{g}{\hat{\theta }}_{n}\in G & \geq & P_{g}\left(A_{n}\left(\theta \right)>\max_{1\leq j\leq k}A_{n}\left(\theta_{j}\right)+\delta ,\;\underset{\left|\right|\theta_{1}-\theta_{2}\left|\right|<\epsilon }{\sup}\left[A_{n}\left(\theta_{1}\right)-A_{n}\left(\theta_{2}\right)\right]\leq \delta \right)\\ & \geq & J_{n,1}-J_{n,2}, \end{array}$$

where

$$\begin{array}{ccc} J_{n,1} & \;:=\; & P_{g}\left(A_{n}\left(\theta \right)>\max_{1\leq j\leq k}A_{n}\left(\theta_{j}\right)+\delta \right),\\ J_{n,2} & \;:=\; & P_{g}\left(\underset{\left|\right|\theta_{1}-\theta_{2}\left|\right|\leq r}{\sup}\left[A_{n}\left(\theta_{1}\right)-A_{n}\left(\theta_{2}\right)\right]\geq \delta \right):=P_{g}\left(\omega (A_{n};\epsilon )\geq \delta \right). \end{array}$$

We now investigate the behavior of $J_{n,1}$ and $J_{n,2}$. Starting with $J_{n,1}$, note that

(93) $$\begin{array}{c} J_{n,1}\geq P_{g}\left(\min_{1\leq j\leq k}\left(A_{n}\left(\theta \right)-A_{n}\left(\theta_{j}\right)-\delta \right)\geq 0\right)=P_{g}\left(D_{n}\geq 0\right). \end{array}$$

Now by (74), it follows that

(94) $$\begin{array}{c} J_{n,1}\geq P_{g}\left(Y_{n}\in B\left(0;r\right)\right), \end{array}$$

where $Y_{n}$ is as in (71) and $r=c_{\theta }\left(\delta \right)$. Applying Proposition 3.4, we obtain

(95) $$\begin{array}{c} \lim_{n\to \infty }\frac{1}{n}\log J_{n,1}\geq -I_{r}\left(\theta \right), \end{array}$$

where $I_{r}\left(\theta \right)=\inf\left\{\Lambda_{\theta }^{*}\left(x\right):x\in R_{\theta }\cap B\left(0;r\right)\right\}$, and we now observe that *r* may be chosen to be $c_{\theta }:=\lim_{\delta ↓0}c_{\theta }\left(\delta \right)>0$, where $c_{\theta }\left(\delta \right)$ is given as in (73). Hence we may replace $I_{r}\left(\cdot \right)$ with I(·) on the right-hand side of the previous equation. Next, using Proposition 4 yields that

(96) $$\underset{n\to \infty }{lim inf}\frac{1}{n}\log P_{g}\left({\hat{\theta }}_{n}\in G\right)\geq \underset{n\to \infty }{lim inf}\frac{1}{n}\log J_{n,1}+\lim_{n\to \infty }\log\left(1-\frac{J_{n,2}}{J_{n,1}}\right)\geq -I\left(\theta \right).$$

Finally, the required lower bound is obtained by maximizing the right-hand side over all θ∈G. □

In the proof of the lower bound, it is clear that the choice of $\left\{\theta_{1},\ldots ,\theta_{k}\right\}$ plays a central role, and the rate function I(θ) will be minimized when *k* is small. As a simple example, suppose that our goal is to obtain a lower bound for $P_{g}\left({\hat{\theta }}_{n}\in G\right)$, where

$$G=\left\{\left(\theta_{1},\theta_{2}\right):\theta_{1}>a_{1}\;\;\mathrm{or}\;\;\theta_{2}>a_{2}\right\}\subset R^{2},\;\theta_{g}\notin G,$$

which is a union of two halfspaces, This can be expressed as a+C, where $a=(a_{1},a_{2})$ and $C=\{\left(\theta_{1},\theta_{2}\right):\theta_{1}>0\;\;\mathrm{or}\;\;\theta_{2}>0\}$, which is an example of a *translated* cone. Now if θ∈G, then we can find two elements which generate the entire set Θ−G, in the sense that all other normalized differences lie between these two unit vectors. These two representative points are the unit vectors $e_{1}=\left(-1,0\right)$ and $e_{2}=\left(0,-1\right)$, and all other normalized differences $(\theta -\tilde{\theta }/∥\theta -\tilde{\theta }∥$ lie between these vectors for all $\tilde{\theta }\in \Theta -G.$ Now going back to (73), we see that this equation again holds. Furthermore, (74) holds with $B(0;c_{\delta }\left(\theta \right))$ now replaced by an intersection of *two* halfspaces rather than of all halfspaces, yielding an unbounded region in the definition of I(θ). This potentially improves the quality of the lower bound compared with what is presented in the statement of Theorem 3. This idea can be potentially generalized to other sets, such as other unions of halfspaces, and so from a practical perspective, could apply somewhat generally.

## 4. Concluding Remarks

In this article, we have derived large deviation results for the minimum Hellinger distance estimators of a family of continuous distributions satisfying an equicontinuity condition. These results extend large deviation asymptotics for *M*-estimators given, e.g., in [6,9]. In contrast to the case for *M*-estimators, our setting is complicated due to its inherent nonlinearity, leading to complications in the proofs of both the upper and lower bounds, and an unexpected subtlety in the form of the rate function for the lower bound. Our results suggest that one can, under additional hypotheses, establish saddlepoint approximations to the density of MHDE, which would enable one to sharpen inference for small samples.

Similar results are expected to hold for discrete distributions. However, the equicontinuity condition is not required in that case, since $\ell_{1}$, unlike $L_{1}\left(S\right)$, possesses the *Schur property*. Hence the LDP in the weak topology of $\ell_{1}$ can be derived (more easily) using a standard Gärtner–Ellis argument, and utilizing this, one can, in principle, repeat all of the arguments above to derive results analogous to Theorems 2 and 3. Large deviations for other divergences under weak family regularity (such as noncompactness of the parameter space Θ)—and their connections to estimation and test efficiency—are interesting open problems requiring new techniques beyond those described in this article.

## Data Availability

Not applicable.
